# Training and assessing undergraduate medical students’ research: Learning, engagement and experiences of students and staff

**DOI:** 10.4102/phcfm.v13i1.2559

**Published:** 2021-01-15

**Authors:** Saajida Mahomed, Andrew Ross, Jacqueline van Wyk

**Affiliations:** 1 School of Laboratory Medicine and Medical Sciences, Faculty of Health Sciences, University of KwaZulu-Natal, Durban, South Africa; 2 School of Nursing and Public Health, Faculty of Health Sciences, University of KwaZulu-Natal, Durban, South Africa; 3 School of Clinical Medicine, Faculty of Health Sciences, University of KwaZulu-Natal, Durban, South Africa

**Keywords:** research skills, undergraduate medical training, education, medical students, South Africa

## Abstract

**Background:**

The development of research skills is an important aspect of undergraduate medical training that facilitates the practice of evidence-based medicine. The inclusion of research training into undergraduate medical curricula can take various formats and is compulsory for all students at the Nelson R. Mandela School of Medicine (NRMSM). The evaluation of this training is important, both to ensure that students obtain the required research skills and to improve the quality of the training.

**Aim:**

The aim of this study was to evaluate undergraduate medical students’ and staff learning, engagement and experiences in the training and assessment of third-year research projects.

**Setting:**

This research was conducted at NRMSM, South Africa.

**Methods:**

Questionnaires were administered to third-year medical students after they completed their research project poster presentations and to the staff who assessed the presentations. Responses to the learning process, group work, alignment between module outcomes and assessment and the benefits of poster presentations were assessed.

**Results:**

A total of 215 students and 10 staff completed the questionnaire. Many students reported having enjoyed learning about research (78%) and that the training activities facilitated their understanding of the research process (84%). The majority of students (86%) and staff (80%) perceived the posters as an effective way to demonstrate students’ ability to collect, analyse and interpret data.

**Conclusion:**

Staff and students viewed the research process positively and reported that the poster presentations were an effective way to assess research.

## Introduction

The development of analytic thinking and research skills in undergraduate medical students is important to increase the awareness and practice of evidence-based medicine in future healthcare practitioners. Higher education institutions in South Africa have been encouraged to find innovative ways to introduce the growing number of undergraduate medical students to basic research. The scholarly role of physicians, both internationally and in South Africa, includes the ability to advance competence-based medical practice through the use and application of scientific and research processes.^1,2^

Globally, the incorporation of research training into undergraduate medical curricula varies from students engaging in elective research activities to their enrolment in mandatory modules.^3,4,5,6^ The benefits of early exposure to research, and its value as an important undergraduate competence that needs to be developed, are widely reported.^3,7,8^ Undergraduate medical students have traditionally not been exposed to or involved in conducting research. Some institutions required interested students to apply to participate in a research programme, which benefited only a few students.^9^ However, given the need to prepare medical students in a more comprehensive way for unknown challenges to be solved in the future, many medical schools have started incorporating research training in undergraduate medical studies.^3^ The experiences from medical schools in both developed and developing countries, such as Pakistan, indicate the importance of longitudinal research training in the scientific practice of medicine.^3,9,10^

The ‘Selective modules’ are part of an innovative, community-orientated primary care approach introduced at the Nelson R. Mandela School of Medicine (NRMSM), KwaZulu-Natal Province, South Africa, in 2002, to develop students’ competence in community-orientated primary care, evidence-based medicine and research methodology. These modules have been described previously.^11^ Selective 01, 02 and 03 modules are offered in the second, third and fourth years of the Bachelor of Medicine and Bachelor of Surgery (MBChB) programme at the University of KwaZulu-Natal (UKZN), in which all medical students are introduced to various aspects of the research process, including epidemiology, study design, ethical approval process, project implementation and ways to disseminate their findings. These modules are incremental and vertically aligned to develop students’ research competence longitudinally over a 3-year period whilst they are enrolled in the 6-year medical (MBChB) programme.^11^

To satisfy the requirements for the research component, all second-year medical students (Selective 01) identify a research topic, based on their observations during a community placement rotation, and are taught how to conduct a literature review on their selected topic.^11^ Building on their experiences in Selective 01, all third-year (Selective 02) students must write a research protocol, submit it for ethical approval to the institutional ethics committee, gather and analyse their data and present their findings in a poster format. Selective 03 (fourth year) students must develop an intervention to address issues identified in their research.

In 2018, 250 students were registered for the Selective 02 module. The students were grouped into 86 groups of two to five students, with each Selective 02 group allocated to a supervisor from Public Health Medicine or Family Medicine. There were nine supervisors for the 86 groups. The supervisor’s role was to guide the students through the process of protocol development and application for ethics approval. This supervision was done via contact sessions and email, and the supervisors provided feedback on the draft protocols and advised their group when the protocol was deemed ready for submission.

The supervision process was supplemented by a series of lectures on research and protocol development. After completion, the draft protocols were marked, feedback was given and the work was returned to the students. Students were then expected to address any deficiencies and submit their updated protocol to the institutional Biomedical Research Ethics Committee (BREC) for ethical permission to conduct the research. Students collected data for their projects in June to July 2018. Following the data collection, students received lectures on data analysis and presenting research findings in a poster format. They met with their supervisors for assistance with the development of the poster. On an annual research day held in September, students present their research findings in a poster format to academic staff involved in teaching and supervision and to peers in their class. Each group was given 10 min to present and 5 min to address questions. A structured marking rubric was used by members of staff to assess each presentation. Each group received verbal feedback at the end of their presentation.

Module evaluations are regularly conducted on all aspects of the MBChB programme for quality assurance purposes and to ensure the alignment of module outcomes and student activities with stated curriculum objectives.^12^ These evaluations provide insight into students’ learning and help to identify areas for modification and additional student support. Various aspects of the three Selective modules have undergone minor amendments since 2009. Whilst the amendments mainly catered for the growing number of students, it was also done to ensure a sustainable and efficient programme to optimise their learning during the process. This article describes the learning, engagement and experiences of students and staff on research training and the assessment of the research projects using a poster presentation.

## Methods

This cross-sectional case study was conducted at UKZN, South Africa, where the NRMSM hosts the undergraduate medical training programme (MBChB). A questionnaire was used to collect data from third-year students who had completed their research module, as well as 10 staff members who served as supervisors or assessors during the research process.

The student questionnaire was divided into five sections, the first four consisting of closed-end questions that required a response selection from five options to enable quantitative analysis. The 22-item questionnaire was developed by the course convener in conjunction with other staff members involved in evaluating the Selective modules. The four sections entailed establishing the students’ responses to (1) the learning process (eight questions), (2) group work (two questions), (3) alignment of poster assessment to module outcomes (four questions) and (4) benefits of posters for assessment and feedback, and students’ preference for a written report rather than poster presentation (eight questions). The last question in the section on poster presentations required a yes or no response on whether the student preferred an alternative method to the poster presentation. Those students who responded ‘yes’ were asked to suggest an alternative assessment method. The final section of the questionnaire was an open-ended question that asked students to comment or make suggestions on any aspects of the poster presentation.

The 16-item staff questionnaire consisted of four sections that used closed-end questions to establish their responses to (1) learning opportunities for staff (three questions), (2) the value of student group work (three questions) and (3) the benefit of posters for assessment and feedback (10 questions). The last question in the third section required a yes or no response to whether the research process could be assessed more efficiently in a different format. Staff who answered ‘yes’ were asked to provide suggestions. The final section of the questionnaire was an open-ended question that asked staff to comment or make suggestions on any aspects of the poster presentation.

Both questionnaires used a 5-point Likert scale to record participants’ responses to the closed-end question items, with ‘5’ indicating strong agreement and ‘1’, strong disagreement to a statement. Although the questionnaires were not piloted, similar questionnaires have been used to assess staff and first-year medical students’ perceptions on the use of posters for teaching and assessment and were found to be suitable as data collection instruments.^13^ The questionnaires were administered immediately after the students had presented their research posters for assessment, whilst the staff completed their forms at the end of the poster assessment session. Data were collected in September 2018.

Each completed questionnaire was numbered individually, and the data were entered into SPSS Version 25 on a password-protected computer by a research assistant. The researchers validated the entries prior to the analysis process. For the purposes of reporting, response categories 5 and 4 (strongly agree and agree, respectively) were combined to indicate agreement with a statement, and categories 1 and 2 (strongly disagree and disagree, respectively) were combined to indicate disagreement. The quantitative responses were analysed by summarising frequency distributions. The open-ended qualitative comments were extracted verbatim and categorised for reporting.

### Ethical consideration

The study was approved by the Biomedical Research Ethics Committee at the University of KwaZulu-Natal (R201/04). All participants signed an informed consent form prior to participating in the study. Potential participants were assured that they would not be penalised if they did not participate or if they withdrew from the study. The questionnaires were completed anonymously, and students were made aware that their responses would not influence the marks allocated for their poster presentation.

## Results

### Respondents’ profiles

Of the 251 students registered for third year, 215 (85.7%) completed the questionnaire, the mean age being 22 years (standard deviation, 2), ranging from 19 to 35 years. The students were from a range of school types, with approximately 28% being from lower quintile schools (one and two), which have limited resources and may not provide students with environments that encourage critical thinking and research. Of the 13 staff members involved in the assessment, 10 completed the questionnaire, the remaining three being investigators in this research. The staff members represented a range of disciplinary and educational backgrounds, including Public Health Medicine, Family Medicine and Clinical Medicine. Their teaching and supervising experience at a higher education institution ranged from being a novice to 10 years.

### Student questionnaire findings

The findings from the students’ questionnaires are given in Table 1. Regarding students’ responses to the learning process, the majority (78.3%) enjoyed learning about research and gained an improved understanding of their research topic from the process of protocol development (84.1%), and reported having learnt from applying for ethical approval (78.9%). Just over three-quarters (76.6%) reported that preparing for the poster presentation had led to an increased engagement with the course material, and 72.1% thought it likely that they would use the research skills in their future careers.

**TABLE 1:** Students’ responses to research training and poster presentation, Nelson R. Mandela School of Medicine, 2018.

Questions	Agree		Neutral		Disagree	
	*n*	%	*n*	%	*n*	%
**Learning process**						
1. I enjoyed learning how to do research in Selective 02 (*n* = 215)	168	78.3	33	15.2	14	6.5
2. Writing a research protocol helped me understand the process of conducting research (*n* = 214)	180	84.1	30	14.0	4	1.9
3. Writing the introduction of the research protocol helped me better understand my research topic (*n* = 214)	181	84.6	26	12.1	7	3.3
4. Writing the literature review for the research protocol helped me better understand my research topic (*n* = 214)	178	83.2	31	14.5	5	2.3
5. Sufficient guidance/support was provided in the writing of the protocol (*n* = 215)	163	75.8	36	16.7	16	7.4
6. Completing the BREC application was a good learning opportunity (*n* = 213)	168	78.9	28	13.1	17	8.0
7. The poster preparation motivated me to engage with the learning material (*n* = 214)	164	76.6	37	17.3	13	6.1
8. I am likely to use the research skills learned in Selective 02 later in my career (*n* = 215)	155	72.1	38	17.7	22	10.2
**Group work**						
1. The poster preparation and presentation encouraged interaction with my group (*n* = 214)	177	82.7	26	12.2	11	5.1
2. Working in a group helped me to learn more than I would have done on my own (*n* = 214)	174	81.3	21	9.8	19	8.9
**Benefit of posters for assessment and feedback**						
1. The poster was an effective way to show that I can collect, analyse, interpret and present data (*n* = 213)	184	86.4	22	10.3	7	3.3
2. The poster was a fair way to assess my ability to collect, analyse, interpret and present data (*n* = 213)	173	81.2	29	13.6	11	5.2
3. Posters are an effective way for academic staff to validate my engagement with the research process (*n* = 212)	166	78.3	39	18.4	7	3.3
4. The peer assessment of posters was beneficial to my learning (*n* = 213)	153	71.8	37	17.4	23	10.8
5. The interaction with the assessor and peers during the poster presentation provided opportunities for meaningful feedback (*n* = 210)	169	80.4	34	16.3	7	3.3
6. Posters are a more efficient way of assessing this research than a written group report (*n* = 214)	170	79.4	29	13.6	15	7.0
7. I would prefer to submit an individual written report instead of the group poster (*n* = 213)	85	39.9	26	12.2	102	47.9


In terms of responses to group work, 82.7% reported that the process encouraged their interactions with the group members, and a similar number (81.3%) agreed that they had benefited from working in a group.

With regard to the benefit of posters for assessment and feedback, three-quarters (77.4%) appreciated the assistance they received from their supervisors and over 80% agreed that the poster presentation was an effective and fair way to demonstrate their research ability. Most (78.3%) agreed that the poster presentation was an effective way for their work to be validated by staff, and 71.8% agreed that their peer assessment was beneficial to their learning. Many (80.4%) found the interaction during the poster presentation to be beneficial, and that posters were a more useful way to assess their research than a written group report. Just over a third of the students (39.9%) indicated that they would have preferred to give an individual written report rather than participate in a group poster. However, of the 134 students who responded to the question on whether they would prefer an alternative method of assessment, only 15 (11.2%) indicated in the affirmative.

**FIGURE 1:**
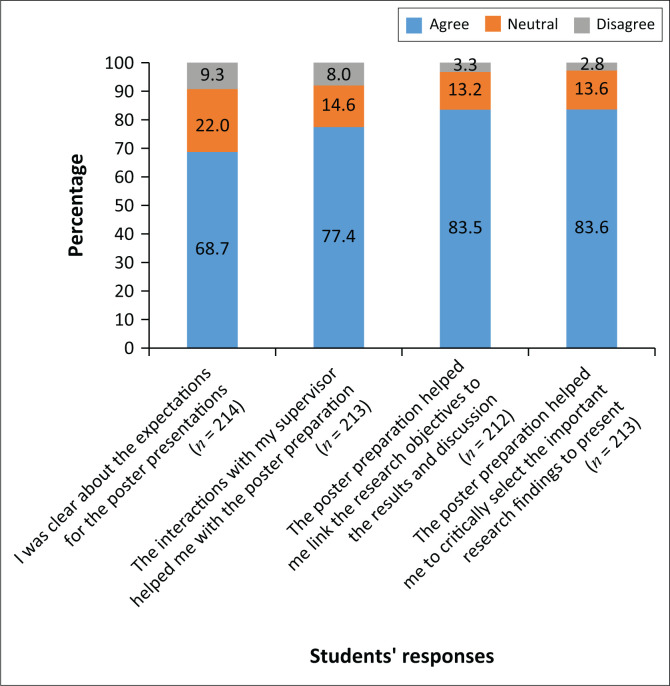
Students’ responses regarding the alignment between the poster presentation and the module outcomes.

With regard to aligning the poster assessment to the module outcomes, only 68.7% agreed that there was clarity about the poster presentation expectations, whilst over 80% agreed that the poster preparation helped them to select the important research findings and to link the objectives to the results and discussion (Figure 1).

### Staff questionnaire findings

In response to the statement probing staff about their learning during the process, 90% of the staff reported that they had benefited from being paired with another assessor. The majority (80.0%) also agreed that the poster assessments provided new insight into the scope of the students’ research (Table 2).

**TABLE 2:** Staff responses to research training and poster presentation.

Questions	Agree		Neutral		Disagree	
	*n*	%	*n*	%	*n*	%
**Learning process**						
1. Being paired with another assessor was a learning opportunity for me (*n* = 10)	9	90.0	1	10.0	0	-
2. Being part of the poster assessment team made the task of assessment enjoyable (*n* = 10)	9	90.0	1	10.0	0	-
3. The poster presentations allowed me to gain new insights into the scope of student community projects (*n* = 10)	8	80.0	2	20.0	0	-
**Group work**						
1. Group supervision was an efficient way to ensure that students achieved the research objectives of Selective 02 (*n* = 9)	8	88.8	1	12.2	0	-
2. In my opinion the group work on the poster supported individual students to learn more than if they had worked alone (*n* = 10)	9	90.0	1	10.0	0	-
3. The poster presentation demonstrated that students interacted within their groups (*n* = 10)	9	90.0	1	10.0	0	-
**Benefits of posters for assessment and feedback**						
1. Through the poster presentation students demonstrated an understanding of the link between the research objectives, results and discussion (*n* = 9)	8	80.0	1	10.0	0	-
2. The poster presentation was an effective way to assess students’ ability to collect, analyse, interpret and present data (*n* = 10)	8	80.0	2	20.0	0	-
3. The posters are a fair method to assess each student’s ability to collect, analyse, interpret and present data (*n* = 9)	6	66.7	2	22.2	1	11.1
4. The poster presentation was an effective way for academic staff to validate student engagement with the research process (*n* = 9)	9	100.0	0	-	0	-
5. The poster presentation was an effective way to assess whether students were able to critically select the important research findings to present (*n* = 9)	9	100.0	0	-	0	-
6. The poster presentations were an efficient way to assess the work of approximately 250 students (instead of marking written reports) (*n* = 10)	8	80.0	2	20.0	0	-
7. The question and answer session provided a good opportunity to give and receive meaningful feedback (*n* = 10)	8	80.0	1	10.0	1	10.0
8. Sufficient time was allocated for each poster presentation (*n* = 9)	7	77.8	2	22.2	0	-
9. The marking rubric was appropriate to assess the poster presentation (*n* = 10)	7	70.0	2	20.0	1	-

With regard to group work, most staff members (88.8%) agreed that group supervision had been effective and that students had learnt more in a group than they would have done if they had been working on their own (Table 2).

Regarding the benefits of posters for assessment and feedback, the majority of staff (80%) agreed that the poster presentation was an effective and efficient way to assess the work of a large student class (Table 2). In addition, 90% agreed that the poster presentation and interaction with the students provided an opportunity for student feedback, to validate that the students actually conducted the research themselves. Six staff members responded to the question enquiring about what we could implement as a more efficient method of assessment. No one indicated that another assessment format would have been more efficient to assess a large number of students’ competence in research.

**FIGURE 2:**
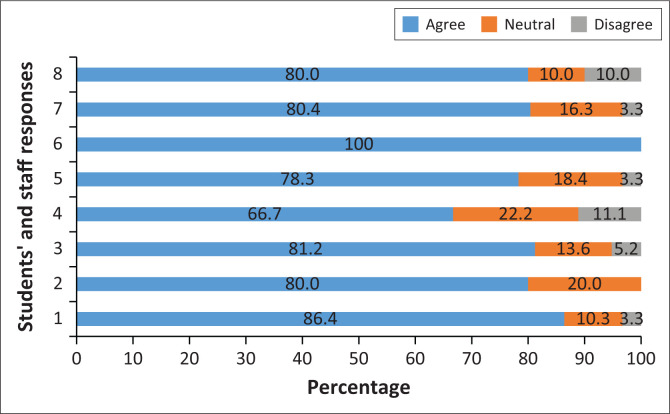
Comparison of student and staff responses on the value of poster for assessment and feedback.

In the two data collection tools, there were four parallel questions asked to students and staff members. These questions asked whether the poster presentation provided an opportunity for feedback to the students, whether it provided an opportunity to validate the research process, whether it was perceived as a fair assessment process and whether it was an effective way for students to demonstrate their research competence. The responses from the students and staff members on these questions are compared and presented in Figure 2.

The majority of students and staff agreed that the use of the poster was a fair and effective method to assess students’ research skills (Figure 2). Whilst all staff agreed that the use of posters was also effective in validating the students’ engagement during the research process, only 78.3% of students concurred. The majority of students (80.4%) and staff (80.0%) agreed that the poster assessment provided a good opportunity to give feedback to students.

### Qualitative findings from students’ and staff questionnaires

We analysed 40 comments from students and four comments from staff members that were obtained in response to the open-ended questions. The comments were grouped thematically and reported verbatim in Table 3. In addition to comments about learning, assessment and group work, students also raised issues concerning the assessment venue. Although there were not many comments, the student feedback was generally positive about the learning they had acquired, and they indicated having enjoyed the learning process (‘I love everything about the poster presentation’). There were two student comments that indicated some degree of discontent with group work and having been allocated a group mark.

**TABLE 3:** Students’ and staff comments about the poster presentations.

Student comments	Staff comments
**Logistics**	**Assessment and feedback**
Larger venue please/better venue	The students did not participate effectively in the question and answer part of the presentation
Venues got crowded during presentation, they were too small for the number of people	Possibly [need] an extra area in the marking rubric to assess the flow of ideas, concepts and coherence. Could be assessed on its own
**Assessment**	**-**
This is a good and beneficial way of assessing group research activities	-
**Learning**	**-**
I love everything about the poster presentation	-
I think it is a great opportunity to learn how to conduct research	-
Poster presentation was a learning experience, it has helped in identifying and learning more about components of poster	-
It would be interesting to learn how to publish our results in a local/international journal	-
**Group work**	**-**
Consider grading individuals for their input and presentation	-
Group work allows individuals to be lazy and not to do much for the overall assessment	-

## Discussion

This is the first reported evaluation of both students’ and staff learning, engagement and experiences on research training and assessment in the undergraduate medical curriculum at the NRMSM. There was a good response rate to the survey, with 85% of students and 76.9% (10/13) of staff completing the questionnaires.

Overall, the majority of students viewed the research process positively, including the development of the research protocol, completing an ethics application and presenting the research findings as a scientific poster. This is consistent with the findings from a systematic review of 14 studies assessing medical students’ perspectives towards research that reported that 74% of students viewed the research process positively.^14^ Student enjoyment of the learning process has been shown to enhance learning, both in the medical field as well as in non-health subjects.^15,16^ However, a number of students also indicate being neutral (33, 15.2%) or in disagreement (14, 6.5%) with the statements, suggesting that for a selection of the students, this exercise was either of no interest or did not provide the basics needed for them to engage in the process.

Regarding the group work component, considering the diverse student intake into the MBChB programme, an important strength of this research training is that all students are expected to actively conduct research, rather than simply learn about the research process in a theoretical manner. Based on our findings, group work offered a safe learning space, allowed students opportunities for meaningful conversations with each other and their supervisors, with 81.3% of students indicating that group work helped them learn more than they would have done if they had worked alone. Although the research is compulsory, students have autonomy in selecting their research topic in an area of interest based on their experiences during Selective 01. Autonomy and relatedness have been described as enabling factors in undergraduate research.^17^ Working in a group would also have allowed for collaborative learning, especially from students with previous research experience. In addition, students from less privileged educational backgrounds may have benefited from the experiences of others in their group. However, group work is not for everyone, with just under 40% of the class indicating that they would have preferred not to work in groups. As mentioned in two students’ comments, students’ dissatisfaction with the group work process is likely because of some students not participating adequately. Experiences of inadequate contribution by group members may stem from those with stronger personalities having taken charge of the group project, resulting in the suppression of the opinions of other students.

More than two-thirds of students reported the likelihood of using their research skills later in their career. This foundation of good research skills developed at an undergraduate level is important, as research has been instituted as a pre-requisite for all specialty-training programmes in South Africa,^18^ with many registrars struggling with this component because of not having had previous research instruction or experience. The findings of this study are in keeping with studies that suggest that early exposure of medical students to practical research activities improves their attitude towards research and scholarship, and that this exposure during training increases the likelihood of medical graduates pursuing related careers and becoming physician scientists.^8^

The focus on developing analytical and research capacity in graduates also helps to improve their preparation for practice, as they are better able to analyse the needs of the patients and communities they serve.^19^ It is also consistent with the competencies expected of graduates from competency-based curricula, which typically consider the design, implementation, assessment and evaluation of medical education programmes when reviewing students’ achievement.^20^

Alignment of learning objectives, content and poster assessments to the research module outcomes is essential to ensure successful teaching and learning.^21^ The majority of students indicated that the preparation of the poster had allowed them to link the various aspects of the research process to the objectives of the module. It was disturbing that only 68.7% of students indicated that they had clarity on the expectations of the poster presentation, despite having had a lecture on poster presentation, the opportunity to meet with their supervisor and the provision of a marking rubric to ensure that they understood the criteria for each section of the poster. The rubric also served to standardise the assessment process amongst the assessors. This finding could stem from students’ over-reliance on their supervisors to emphasise important issues, a reflection on the type and quality of supervision provided or may indicate a lack of student engagement with resource material provided. Further study is needed to explore this issue.

In terms of the benefits of the posters for assessment and feedback, the majority of students and staff indicated that this was an efficient and effective method to present research findings. Our findings further affirm the value of posters in the assessment process as preparing and marking written research reports for this number of students would have been extremely time-consuming for students and staff, respectively. There is also a limited number of staff available to supervise and mark the projects. It would have been difficult and stressful to complete the projects and the assessment of written assignments within the same academic year. In addition, the majority (78.3%) of students and all the staff felt that the poster presentations were an effective way to validate the work of the students.

Plagiarism is a major problem at UKZN and other universities around the world, with UKZN having a no-tolerance policy towards it.^22^ In our module, plagiarism is addressed with the students prior to submission of their research protocols. Students sign a declaration stating that their work has not been plagiarised and the research protocols undergo a similar check using Turnitin. Meaningful interaction with students, although challenging in large classes, offers academic staff the opportunity to validate students’ submissions by probing and asking questions about their work, as was done at the poster presentations. It was encouraging to note that the majority of both staff and students felt that the poster presentations were a useful way to validate that the work submitted was done by the students.

The successful use of posters for assessment has been reported amongst staff and first-year students at NRMSM, and in various undergraduate disciplines at other institutions.^13,23^ Interestingly, 40% of students indicated a preference for an individual report, which may be because of individual students’ dissatisfaction with the allocation of a group mark. There was, however, a discrepancy in this finding, as only 11% of students responded positively when another item of the same questionnaire prompted whether they would prefer an alternative method to the poster presentation. This inconsistency warrants further investigation.

With regard to the assessment process, the majority of staff indicated that the time for the poster presentation was adequate for the students to cover all aspects of their work and for them to record a mark and give feedback. The marking rubric is revised annually in an attempt to make it user-friendly and concise. However, one of the comments from a staff member was for the rubric to assess flow of ideas, concepts and coherence independently as these aspects are currently being assessed as part of the poster content. It is possible that the students’ oral presentations are therefore not aligned to the way in which the poster is written. The value of online and visual representations in mapping curricular links to learners has been reported,^24^ which may be an avenue to explore in the future.

In addition to the efficiencies of the poster presentation, the session also allowed for a collegial atmosphere in which both staff and students learnt. The diverse background of staff as well as their varying years of experience in research may have facilitated peer learning. However, a few students did note their dissatisfaction with the venue for the poster presentations, which took place in a large multi-purpose hall at the university, which was the only venue available for such an exercise. The posters were displayed on boards and groups of students present to examiners simultaneously, which can present a challenge in terms of noise levels and distractions. Although nearly 80% of students and staff viewed the question and answer session positively, one staff member commented that students did not participate effectively during these sessions, which is possibly because of their limited experience of conducting and engaging with research.

The limitations of our study are inherent to its case study methodology, this being a single cohort of students without a comparison with other methods of research training and assessment. We did not assess the dynamics of group work, such as managing conflict, unequal distribution of work, the extent of individual student’s contribution to the final poster and how the students managed their time. By their very nature, student self-evaluations are limited by their level of knowledge and understanding of the aspects involved in assessing a complex skill.^25^ In addition, the timing and administration of the questionnaire after the student poster sessions, may have introduced a bias, as students may have perceived some advantage in obtaining good marks, if they provided positive responses.

Although the findings may have limited generalisability because of the unique context, we believe that our strategy for teaching and assessing research skills in undergraduate medical students can be adapted to many other settings, including other fields in health sciences. As results were anonymous, we could not link the findings to specific students or school quintiles, to assess whether students from the lower quintile schools possibly require more intensive support with the research process. Whilst students had the opportunity to make additional comments at the end of questionnaire, the majority did not do so. We were therefore unable to interrogate the reasons where students had disagreed with statements. These are issues that require further investigation and qualitative follow-up.

Future research needs to focus on how to improve our communication about the requirements for the poster presentation. In addition to the ongoing evaluation of the module, we advocate for follow-up research on these students as practitioners, to assess the long-term benefits of the research training.

## Conclusion

Both staff and students perceived the compulsory research training in the undergraduate medical curriculum positively. Students reported that the research training improved their collaborative learning and engagement with learning material. Resource-constrained institutions with large student numbers make it difficult to operationalise the teaching and assessment of the research component at the undergraduate level. This article demonstrates that the poster presentations were effective and efficient for assessing the research of a large cohort of students and provided an enabling environment for learning amongst staff and a diverse student population.
